# HBV Reactivation in Immunosuppressed Patients: Screening, Prevention, and Management Including Solid Organ Transplant Recipients

**DOI:** 10.3390/v17030388

**Published:** 2025-03-09

**Authors:** Philip Vutien, Mindie H. Nguyen

**Affiliations:** 1 Division of Gastroenterology and Hepatology, University of Washington Medical Center, 1959 NE Pacific Street, Box 356175, Seattle, WA 98195, USA; 2 Division of Gastroenterology and Hepatology, Stanford University Medical Center, Palo Alto, CA 94305, USA; mindiehn@stanford.edu; 3 Department of Epidemiology and Population Health, Stanford University Medical Center, Palo Alto, CA 94305, USA

**Keywords:** hepatitis B, de novo infection, immunosuppression, chronic hepatitis B infection

## Abstract

Hepatitis B virus (HBV) infection remains a global health challenge, affecting over 254 million individuals chronically and contributing significantly to cirrhosis, liver failure, and hepatocellular carcinoma. Despite advancements in antiviral therapy, HBV reactivation remains a critical concern, particularly in immunosuppressed individuals, including non-transplant patients undergoing immunosuppressive therapy and solid organ transplant recipients. This review provides screening and management strategies for HBV reactivation in these populations.

## 1. Introduction

Globally, more than 2 billion people are estimated to have been exposed to the hepatitis B virus (HBV), and approximately 254 million are chronically infected, making chronic hepatitis B (CHB) one of the leading causes of cirrhosis, liver failure, and hepatocellular carcinoma (HCC) [1]. Viral suppression with nucleos(t)ide analogs (NA) dramatically reduces the risks of these adverse liver-related outcomes, yet there are currently no curative therapies [2]. As a result, individuals with chronic hepatitis B (HBV surface antigen [HBsAg] positive) and those who were previously infected (HBsAg negative/core antibody [anti-HBcAb] positive) remain at risk for HBV reactivation [3]. Although HBV reactivation can occur spontaneously, this risk is significantly increased with exposure to immunosuppressive therapies. In this review, we will provide an overview of managing and mitigating the risk of HBV reactivation in two clinical scenarios: (1) non-transplant patients on immunosuppressive medications and (2) solid organ transplant recipients.

## 2. The Pathophysiology and Clinical Course of HBV Reactivation

HBV is a hepatotropic virus that can be transmitted via blood, semen, and other body fluids from an infected individual [1,4]. In patients with chronic HBV infection, viral control is the result of a complex interaction between virologic factors and host immunity. While many patients with chronic HBV infection may have an immune-active disease warranting antiviral treatment, some may have an inactive disease (e.g., low HBV DNA level, normal liver enzymes, and histology), which portends a low risk of disease progression; thus, long-term antiviral treatment is not recommended [2,5]. However, these patients with immune-inactive chronic HBV infection may still reactivate with a rapid rise in HBV DNA levels and/or liver enzymes either spontaneously or with immunosuppressive therapy [6,7,8,9]. For those with CHB (HBsAg positive), HBV reactivation is defined as any one of the following: (1) a 100-fold increase in HBV DNA compared to the baseline level; (2) HBV DNA ≥ 10,000 IU/mL if the baseline HBV DNA level is unknown; or (3) HBV DNA ≥ 1000 IU/mL in a patient with a previously undetectable level [10].

Even those with resolved HBV infection are at risk for HBV reactivation due to the persistence of covalently closed circular DNA (cccDNA) formed within the nucleus of the hepatocytes previously infected with HBV, which provides a template for future HBV replication [11]. These patients can thus be described as having “latent HBV”, as indicated by an HBsAg-negative/HBVcAb-positive serostatus. For patients with latent HBV, HBV reactivation is defined by de novo HBV replication as confirmed by new HBsAg positivity and/or a newly detectable HBV viral load [12].

Once HBV reactivation occurs, the clinical course varies considerably, but some will experience life-threatening complications, including liver failure, with high mortality since many of these patients taking immunosuppressive therapies are not candidates for rescue liver transplant due to their oncologic or non-oncologic comorbidities. HBV reactivation in the context of immunosuppression presents initially with a marked increase in HBV DNA levels. This phase can start as early as within a few weeks after immunosuppression initiation, and patients are often asymptomatic. Among those with HBV reactivation, up to 40% will develop HBV reactivation-related hepatitis [6,7,13], as characterized by elevated alanine aminotransferase (ALT) and aspartate aminotransferase (AST) levels. This hepatitis phase lags behind the HBV replication phase by several weeks. Although not all patients who experience HBV reactivation will develop reactivation-related hepatitis, the presentation can be severe, manifesting in jaundice, liver synthetic dysfunction, and even liver failure requiring salvage liver transplantation despite the use of NA therapy [14]. Furthermore, late reactivation has been described as occurring even beyond 12 months after cessation of immunosuppression due to a delay in immune reconstitution [8].

## 3. Screening and Testing for HBV

**Figure 1:**
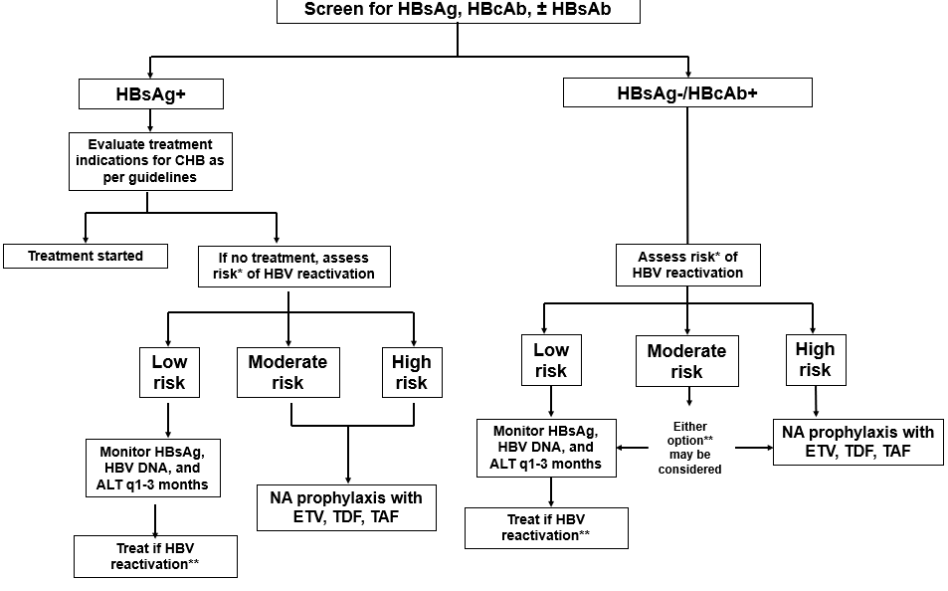
Algorithm for the management of HBV reactivation based on HBV serology and risk categories for patients receiving immunosuppression. * HBV risk is defined as (1) low risk: <1% risk of reactivation; (2) moderate risk: 1–10% risk of reactivation; (3) high risk: >10% risk of reactivation. ** NA treatment should consist of entecavir (ETV), tenofovir disproxil (TDF), and tenofovir alafenamide (TAF).

Professional society management guidelines from a variety of medical subspecialties have uniformly recommended HBV screening (HBsAg and anti-HBc) prior to initiating immunosuppressive therapy (Figure 1) [2,5,10,12]. However, universal HBV screening inclusive of HBsAg, anti-HBc, and anti-HBs has been recommended by the U.S. Centers for Disease Control (CDC) since March 2024 for all U.S. adults [15]. A positive serologic marker (HBsAg and/or anti-HBc) should be followed up with an HBV DNA viral level. Hepatitis B surface antibodies can also be assessed, and patients are offered vaccination if non-immune. In clinical practice, patients who are identified to have CHB (HBsAg positive) should undergo a complete evaluation, including baseline HBV viral load, ALT level, HBeAg and HBeAb status, serologic evaluation for co-infections (HIV, HCV, HDV), and an assessment of underlying fibrosis to determine any indications to start NA therapy regardless of any planned immunosuppressive therapy. The indication for antiviral therapy in CHB outside the setting of immunosuppressive therapy is beyond the scope of this review but has been addressed by practice guidelines [2,10].

Despite these recommendations, real-world screening rates are suboptimal and highlight a critical deficiency in HBV screening prior to the initiation of immunosuppressive therapies. In a U.S. multi-institutional study of 11,959 oncologic patients who were planning to receive immunosuppressive therapies as part of their cancer treatments, only 2045 (17.1%) were screened for either HBsAg or anti-HBc [16]. Among those tested, 0.9% had CHB (HBsAg positive), and 8.4% were previously exposed (HBsAg negative/anti-HBc positive). In another cross-sectional study of national claims data from Japan, among 82,282 patients with rheumatoid arthritis, only 9.7% had received the appropriate HBV screening prior to initiation of immunosuppressive therapy [17]. These findings highlight a critical deficiency in HBV screening practices and a need for patient and provider education, as well as changes in system practice.

In addition to conventional HBV serologies and standard HBV DNA PCR testing, several newer HBV biomarkers are available for use. Ultra-sensitive HBV DNA [18] testing may detect very low-level viremia (beyond standard HBV DNA testing), which may help to identify those at higher risk for reactivation. Quantitative HBsAg (qHBsAg) [19] has also been found to be more sensitive than standard HBV DNA in reflecting underlying HBV disease activity. Other emerging biomarkers include hepatitis B core-related antigen [20], HBV pregenomic ribonucleic acid [21] (both may serve as surrogates for cccDNA activity), and quantitative anti-HBc [22] (lower levels may be associated with a higher risk of HBV recurrence after liver transplantation). It remains unclear whether these biomarkers provide significant incremental benefits over routine HBV serologic testing and then standard HBV DNA monitoring in most clinical scenarios.

## 4. Risk Factors for HBV Reactivation and Clinical Presentation

HBV risk stratification is crucial for guiding subsequent management strategies. A key determinant of reactivation risk is the HBV serologic status: patients with CHB (HBsAg positive) have a significantly higher risk of reactivation compared to those with latent HBV (HBsAg negative/anti-HBc positive) [12,23]. For example, a meta-analysis of 29 studies involving 1409 HBV-infected patients found that the prevalence of HBV reactivation among CHB patients receiving biologic therapies ranged from 17.1% to 40.5% [24]. By contrast, the reactivation rate among those with occult HBV infection ranged from 2.6% to 6.4%.

Consequently, subsequent management strategies are further tailored according to whether the patient has CHB or latent HBV. Within each HBV status, patients can be categorized into three risk categories for reactivation and with additional recommendations for management changes: low risk (<1%), moderate risk (1–10%), and high risk (>10%) for reactivation (Figure 1) [5,12,14]. Beyond HBsAg and anti-HBc status, other virologic characteristics associated with increased HBV reactivation risk include high baseline viral load [25], non-A HBV genotype [26], HBeAg seropositivity [25], and the absence of anti-HBs among patients with latent HBV [27].

### 4.1. Risk Stratification According to Type of Immunosuppression

Risk stratification is further guided by the type of immunosuppressive therapy that is administered (Figure 1, Table 1). One of the most well-characterized is B-lymphocyte-depleting therapies, such as rituximab, which carries a “black box” warning for HBV reactivation. Rituximab is an anti-CD20 monoclonal antibody rituximab which depletes B-lymphocytes through immune-mediated mechanisms. For both patients with CHB and latent HBV, the risk of reactivation has been consistently shown to be within the “high risk” (>10%) category [9,28]. In addition, a substantial proportion of HBV reactivation occurs even 12 months after discontinuation of rituximab, presumably due to delayed immune recovery [28,29]. As such, major societies have advised continuing NA therapy for 12–18 months after the completion of rituximab or similar agents (i.e., rituximab, ofatumumab, obinutuzumab) [2,5,10,12].

Therapies associated with a low risk of reactivation for patients with either CHB or latent HBV include brief (<1 week) courses of corticosteroids, antimetabolites/antiproliferative agents (e.g., azathioprine, 6-mercaptopurine, methotrexate, mycophenolate mofetil), and intra-articular steroid injections. Other therapies including high-dose corticosteroids, anthracyclines, TNF-alpha inhibitors, cytotoxic chemotherapies, and calcineurin inhibitors are associated with either a moderate (1–10%) or high risk (>10%) of HBV reactivation, depending on whether they are used in patients with CHB or latent HBV. We refer the readers to other publications for a more detailed discussion regarding HBV reactivation risk associated with each type of immunosuppressive therapy [5,12,31,32].

### 4.2. Patient and Disease Characteristics Associated with HBV Reactivation

In addition to HBV serology and the type of immunosuppressive treatment, several patient-related factors can also impact the risk of HBV reactivation. Patient characteristics associated with a higher risk of HBV reactivation include male sex, older age, advanced fibrosis, and hematologic (vs. non-hematologic) disorders. Among patients with cirrhosis, the risk of HBV reactivation is notably higher (ref), and the consequences are more severe. Cirrhotic patients face increased rates of hepatitis, liver failure, and mortality should HBV reactivation occur. A meta-analysis performed by Cholongitas et al. found that patients with hematologic malignancies were at a higher risk of HBV reactivation (10.9%) when compared to patients with non-hematologic diseases (3.6%) [23].

Co-infection with other hepatotropic or non-hepatotropic viruses can pose special clinical scenarios. In patients with chronic hepatitis C virus (HCV) infection and HBV co-infection, the use of direct-acting antiviral therapy has been linked to the risk of HBV reactivation. A recently published systematic review found that the baseline risk of reactivation was low (2 per 1000) for patients with latent HBV but high (240 per 1000) for patients with CHB [3]. Co-infection with HBV/human immunodeficiency virus (HIV) is also commonly seen due to the shared routes of transmission. Current guidelines recommend initiating antiretroviral therapy in all HIV-infected individuals—regardless of CD4 count—which includes agents with anti-HBV activity, typically tenofovir (TDF or TAF) plus emtricitabine or lamivudine [33]. Treatment interruptions should be avoided, as they can lead to HBV reactivation and severe hepatitis. If a change in the HIV/HBV regimen is required due to HIV virologic failure or other reasons, HBV-active agents must be maintained or replaced with equally potent alternatives, considering potential HBV cross-resistance (e.g., prior LAM exposure can lead to an increased risk of ETV resistance). For more comprehensive discussions on managing HIV/HBV co-infection, readers are referred to published guidelines and reviews on this topic [34].

## 5. Risk-Stratified Approach to Managing HBV Reactivation

Two management strategies are employed to manage the risk of HBV reactivation. The first strategy is to initiate NA prophylactic therapy during and for 6–12 months following the end of immunosuppressive treatments. The second strategy is to monitor HBV studies (ALT, HBsAg, HBV DNA every 3 months) and provide “on-demand” NA therapy at the first sign of HBV reactivation. When deciding between antiviral prophylaxis versus monitoring, shared decisions and discussions should be made between patients and providers to realistically evaluate patient ability and willingness to maintain regular long-term monitoring, as often required for patients receiving immunosuppressive therapies for non-oncologic conditions.

For those with CHB, exposure to a majority of immunosuppressive therapies leads to a moderate (1–10%) to high (>10%) risk of HBV reactivation. For these patients at moderate and high risk of HBV reactivation, the prophylactic NA strategy should be considered. Data from randomized control and prospective studies have demonstrated that prophylactic NA therapy can dramatically reduce, up to five-fold, risks of HBV reactivation and HBV reactivation-associated hepatitis [35,36,37]. For patients at low risk of reactivation, serial monitoring with on-demand NA therapy is recommended.

For patients with latent HBV, prophylactic NA therapy is recommended for those at high risk for reactivation, including patients with non-Hodgkin’s lymphoma who are exposed to rituximab or similar B-cell-depleting agents. Either strategy of NA prophylaxis or monitoring with on-demand NA therapy can be employed for patients at moderate risk (1–10%), with the choice of either strategy tailored to the patient’s comorbidities, history of adherence, and intended duration of immunosuppressive therapy. Patients with latent HBV and at low risk (<1%) of reactivation should undergo serial monitoring with on-demand NA treatment if there is evidence for reactivation. In situations where an on-demand strategy is limited by the unavailability or prohibitive cost of repeated HBV DNA testing, a prophylactic approach with upfront therapy is advisable.

An important monitoring and treatment principle to take note of is the potential changes in patient immunosuppressive regimens, which frequently occur due to changes in the underlying disease. For example, patients with a low-risk treatment may have progression of the primary disease, prompting an increase in their steroid doses, changes to a high-risk category therapy, and/or a combination of therapies that would now place the patients at high risk for HBV reactivation for which prophylactic antiviral would become indicated.

Finally, if prophylactic antiviral treatment is initiated, medications with high potency and high genetic barrier first-line NA therapy, such as entecavir (ETV), tenofovir disproxil (TDF), or tenofovir alafenamide (TAF), should be used. It is also imperative to continue prophylactic antiviral treatment until at least 6–12 months after the end of the immunosuppressive medications, except for those with B-cell-depleting biologics when a longer course (18 months) may be prudent due to reports of delayed to very delayed reactivation with these agents.

## 6. Treatment of Reactivation

All patients with HBV reactivation should be treated with first-line NA therapy, such as ETV, TDF, or TAF. These agents are preferred over older ones, such as lamivudine, telbivudine, and adefovir, since there is an increased risk of developing a drug-resistant virus with these agents. For those early in their HBV reactivation, the goal is to prevent progression towards severe hepatitis and/or hepatic failure, which can occur in up to 40% of patients who experience HBV reactivation [13]. The decision to interrupt immunosuppressive therapy should be individualized based on the patient’s clinical presentation. Patients with a mild, isolated elevation in HBV DNA levels may be monitored on continued immunosuppression, particularly if NA therapy is initiated promptly. However, those with reactivation-associated hepatitis may require temporary reduction or cessation of immunosuppressive therapy until there is adequate suppression of their HBV viral load and a significant decrease in their liver enzymes.

## 7. HBV in Solid Organ Transplant Recipients

As with non-transplant settings, patients with CHB and latent HBV who are solid organ transplant recipients may be at risk for HBV reactivation due to the lifelong immunosuppression (e.g., corticosteroids, lymphoid-depleting agents, calcineurin inhibitors) required to prevent allograft rejection.

In addition to recipient-related HBV reactivation, there is an added consideration of donor-derived HBV infections. As of 1 March 2021 per U.S. organ procurement organization policy, all potential donors are tested with HBsAg, anti-HBc, and HBV DNA to identify those with CHB or latent HBV. For HBV-negative recipients, the use of these allografts from HBsAg-positive and/or anti-HBc-positive donors confers risk of de novo HBV infection to the recipient, requiring tailored recipient–donor matching, prophylactic strategies, and post-transplant monitoring. This section will focus on screening, risk stratification, and management strategies for HBV in solid organ transplant (SOT) recipients. For a discussion of HBV reactivation in hematopoietic stem cell transplant recipients, we direct the readers to these other reviews [38,39,40].

Post-transplant HBV serologic monitoring for SOT recipients deserves special attention. As the natural course of HBV infection is influenced by both viral replication and host immunity, HBV control in the post-transplant setting is heavily influenced by the degree of immunosuppression. As with the risk of post-transplant infection with Epstein–Barr virus and other opportunistic pathogens, the risk of HBV recurrence or reactivation escalates with higher degrees of immunosuppression [41]. Therefore, patients should be monitored more closely during periods of more intense immunosuppression (i.e., the first year post-transplant) with laboratory studies that include HBsAg and HBV viral load. If NA therapy fails to fully suppress the HBV DNA level, further evaluation with HBV DNA polymerase testing should be performed to guide tailored adjustments to the treatment regimen. Additional strategies to achieve complete viral suppression should be considered if not achieved with a single agent. These strategies may include combination therapy with two NAs, such as tenofovir/emtricitabine or tenofovir/entecavir. The tenofovir/emtricitabine combination offers a lower-cost, single-pill option, while tenofovir/entecavir may provide greater efficacy by combining two first-line agents.

### 7.1. Solid Organ Transplantation in Recipients with CHB

Prophylactic treatment should be used to prevent HBV infection of the liver allograft and post-OLT recurrent infection in the HBsAg-positive recipient/HBV-negative donor (Table 2).

For patients with end-stage liver disease and/or hepatocellular carcinoma due to CHB, an orthotopic liver transplant can be a life-saving intervention. During the transplant surgery, the recipient’s HBV-infected liver is replaced. Yet, there is a risk of HBV recurrence in the transplanted liver allograft due to extrahepatic viral reservoirs in the recipient’s circulation and lymphatic system. Without any prophylaxis, the HBV recurrence rate of HBV has exceeded 80%, resulting in a poor graft and recipient survival rate of only ~50% at 5 years post-OLT [42,43].

However, these outcomes are now of historical significance due to advancements in prophylactic strategies. For OLT recipients with CHB, a combination of NA and HBIG therapy has been proven effective in reducing HBV recurrence to <5% while ensuring excellent graft and recipient survival rates [44]. Nonetheless, maintaining long-term viral suppression is crucial, underscoring the importance of selecting NAs with a high barrier to resistance. Currently, these NAs include ETV, TDF, and TAF. When selecting between these options, it is important to consider common nonhepatic comorbidities in the post-transplant population, such as chronic renal insufficiency and poor bone health. Approximately 20% of transplant recipients develop chronic kidney disease within five years post-OLT, primarily due to calcineurin inhibitors and other nephrotoxic agents [45]. Preliminary evidence suggests that TDF use is associated with higher rates of nephrotoxicity compared to other NAs [46,47,48]; therefore, those with renal dysfunction may benefit from using ETV or TAF long term. Older agents such as lamivudine, adefovir, and emtricitabine are not recommended due to their high rates of drug resistance, which compromise long-term viral suppression. Higher HV viral levels at the time of transplant have been associated with an increased risk of HBV recurrence [49]. Thus, all OLT candidates with CHB should receive suppressive NA therapy, with the goal of achieving as low an HBV viral level as possible and reducing the risk of HBV recurrence post-OLT.

In addition to lifelong suppressive NA therapy, many transplant programs incorporate HBIG therapy to prevent HBV recurrence after OLT [50,51,52]. HBIG blocks HBV nucleocapsid entry into hepatocytes and neutralizes circulating HBV via a recipient antibody-mediated immune response. HBIG protocols differ in dose and duration according to the transplant center. Recipients at low risk for recurrence receive HBIG peri-operatively or within the first week post-OLT. However, recipients with risk factors for HBV recurrence (e.g., elevated pre-transplant HBV viral load, history of non-adherence, and baseline NA antiviral resistance) are typically recommended to receive HBIG infusions up until the first year post-OLT. Those who are co-infected with HDV are also recommended an extended course of HBIG, as HBV recurrence invariably leads to HDV co-recurrence, for which there are limited therapies. However, not all programs utilize HBIG due to the added costs and unclear benefits beyond NA monotherapy alone [44,52,53].

Prophylactic treatment is used to prevent HBV reactivation in HBsAg-positive recipients of nonhepatic solid organs.

For nonhepatic (e.g., kidney, pancreas, heart, lung, etc.) solid transplant recipients with CHB, management focuses on preventing HBV reactivation, as the native HBV-infected liver remains intact (Table 3). Much of the data from nonhepatic SOT recipients are derived from kidney transplant recipients. In renal transplant recipients with CHB, the risk of HBV reactivation without antiviral prophylaxis is markedly elevated, ranging from 50% to 94%, which leads to much poorer recipient survival rates. Thus, to prevent recurrent HBV and its attendant complications, current guidelines recommend lifelong prophylactic antiviral therapy with a high-barrier NA to prevent reactivation and its associated complications. While LAM has historically improved survival in renal transplant recipients, its high rates of resistance limit its long-term use. Thus, high-barrier NAs are preferred due to their long-term efficacy. As with OLT recipients transplanted for CHB, ETV and TAF may be preferred over TDF due to a lower risk of renal toxicity [47,48].

Prior to transplant, all SOT candidates with CHB should be evaluated by a provider with expertise in the management of HBV, and NA therapy should be initiated as per guidelines. If NA therapy is not initiated pre-transplant, it should be started at the time of transplant and continued indefinitely, as the risk of HBV reactivation persists as long as immunosuppressive therapy is required. An exception is if immunosuppression is discontinued following graft failure, such as in the case of kidney allograft failure necessitating a return to dialysis.

### 7.2. Solid Organ Transplantation in Recipients with Latent (Anti-HBc-Positive) HBV

OLT recipients with latent HBV (HBsAg negative, anti-HBc positive)

Recipients with latent HBV and who receive an HBV-negative liver allograft typically do not need antiviral prophylaxis due to a minimal risk of HBV recurrence [54,55]. The native liver, the main source of HBV recurrence, is removed. These patients undergo HBV viral studies (HBsAg, HBV viral load) every 1–3 months for the first year and then annually afterward.

Nonhepatic SOT recipients with latent HBV (HBsAg negative, anti-HBc positive)

In contrast to OLT recipients, nonhepatic SOT recipients retain their native liver and thus have a low, albeit substantial (<5%) risk of HBV reactivation. A systematic review and meta-analysis of 16 retrospective cohort studies and 2913 nonhepatic SOT recipients reported an overall HBV reactivation rate of 2.5% [56]. On subgroup analyses, the reactivation rate was significantly higher in patients who were non-immune (anti-HBs negative; 7.8%) and received lymphoid-depleting therapies (7.3% for recipients who received rituximab, 4.9% for those who received anti-thymocyte globulin (ATG)). Among those with HBV reactivation, complications were frequent and serious; 11% of recipients with HBV reactivation experienced HBV-related graft failure and/or died.

Data regarding the optimal management strategy in this population remain limited. While guidelines generally do not recommend routine prophylactic antiviral therapy [50,54,55], some centers opt for initiating NA prophylaxis in patients with higher-risk profiles, such as those who are anti-HBs negative (non-immune) and/or receiving B-cell depleting therapies, such as rituximab or other lymphodepleting agents, such as alemtuzumab or ATG. In one retrospective cohort study of 180 nonhepatic SOT recipients transplanted at the Mayo Clinic, 77 recipients received prophylactic NA therapy, and 103 recipients did not receive NA prophylaxis [57]. No recipient who received prophylactic NA therapy experienced HBV reactivation. In contrast, 12 of 97 (12%) of those who did not receive prophylaxis experienced HBV reactivation. HBV reactivation occurred in 29% (2/7) of recipients exposed to rituximab and 100% (2/2) who were exposed to lymphoid-depleting agents, such as ATG or alemtuzumab. Thus, given the excellent side effect profile of first-line antiviral agents, such as ETV and TAF, and the availability of low-cost generic ETV, it may be prudent to consider prophylactic antiviral treatment in this population.

### 7.3. Solid Organ Transplantation in HBV-Negative Recipients with Anti-HBc-Positive Allografts: Risk of Graft-Related de Novo HBV Infection

OLT recipients of anti-HBc-positive allografts

The availability of anti-HBc-positive allografts has significantly expanded the donor pool, particularly in HBV-endemic regions, such as Asia and Africa, without compromising recipient or graft survival. While these allografts may be best allocated to recipients with CHB (i.e., recipient HBsAg positive), with the appropriate prophylactic strategies, even HBV-negative (i.e., HBsAg negative) recipients of an anti-HBc-positive liver can achieve excellent outcomes. A retrospective study from Hong Kong described a similar 10-year graft survival rate for 416 recipients of anti-HBc-positive grafts (76.8%) when compared to 548 recipients of anti-HBc-negative grafts (74.8%) and without any difference in graft dysfunction, recipient death, or hepatocellular carcinoma [58].

However, the utilization of these liver allografts does carry a risk of de novo HBV infection to the recipient via transmission from the transplant allograft. The risk depends also on the recipient’s HBV serologies. Without any prophylaxis, recipients who are HBV naïve/non-immune (anti-HBc negative, anti-HBs negative) are at the highest risk for de novo HBV infection (~48%), followed by recipients who are HBV exposed/non-immune (anti-HBc positive, HBsAg negative, anti-HBs negative; risk~13%) and then recipients who are vaccinated (anti-HBc negative, anti-HBs positive; HBV risk~9%) [59]. Recipients who are naturally immune (anti-HBc positive, anti-HBs positive; HBV risk ~1%) have the lowest risk of de novo HBV infection.

Many transplant programs administer prophylactic NA to recipients at higher risk of de novo HBV infection (e.g., all recipients except those naturally immune). In a meta-analysis of 26 studies and 462 recipients of anti-HBc-positive liver allografts, NA prophylaxis substantially reduced the risk of de novo HBV infection from 58% to 11% in HBV-naïve/non-immune (anti-HBc negative, anti-HBs negative), 18% to 2% in vaccinated (anti-HBc negative, anti-HBs positive), and 14% to 3% in HBV-exposed/non-immune recipients (anti-HBc positive, anti-HBs negative) [60]. In this study, NA prophylaxis did not reduce the risk of de novo hepatitis in recipients who were naturally immune (anti-HBc positive, anti-HBs positive). HBIG is not typically utilized as it does not provide additional benefits for preventing de novo HBV infection beyond NA monotherapy in this setting [59].

Nonhepatic SOT recipients of anti-HBc-positive allografts

The risk of transmission for anti-HBc-positive allografts is mainly observed in OLT recipients; nonhepatic SOT recipients have much lower risks of de novo HBV infection (<1%). In a recently published meta-analysis of 13 studies and 2516 recipients of anti-HBc-positive kidney allografts, only nine (0.36%) cases were reported [61]. Notably, the risk of HBV infection was significantly higher among recipients who did not receive prophylaxis and lacked immunity, with a rate of 5.71% (2/35) observed in those who were anti-HBc positive and anti-HBs negative. There were no differences in recipient or graft survival for recipients of anti-HBc-positive vs. anti-HBc-negative kidney allografts. Similarly low rates of HBV infection and excellent outcomes have been reported for the use of anti-HBc-positive thoracic allografts, although data are scarce [62,63,64].

All potential SOT recipients who are HBV non-immune should be vaccinated. The optimal prophylactic strategies for recipients of nonhepatic anti-HBc-positive allografts post-transplant remain uncertain, but based on limited data regarding a higher risk of infection for non-immune patients [61], NA prophylaxis can be considered for those who are HBV non-immune. In the absence of NA prophylaxis, routine post-transplant monitoring, including HBsAg, HBV DNA every one to three months, and on-demand therapy, is recommended.

### 7.4. Solid Organ Transplantation in HBV-Negative Recipients with HBsAg-Positive Allografts: Acquired Chronic HBV Infection

OLT recipients of HBsAg-positive allografts

Liver allografts from donors who are HBsAg positive or HBV nucleic acid testing (NAT) positive are not routinely utilized, as their use typically results in chronic HBV infection in recipients, which invariably results in chronic HBV infection. However, a growing body of literature suggests that HBsAg-positive allografts can effectively expand the donor pool and achieve similar patient and graft outcomes to those of HBsAg-negative ones [65,66,67,68,69]. Ali et al. compared clinical outcomes in 209 OLT recipients of HBV-positive allografts (defined as HBsAg positive or HBV NAT positive) to 1045 matched recipients of HBV-negative allografts using data from the Organ Procurement and Transplantation Network (OPTN) database. This study found no statistically significant differences in recipient mortality (3-year survival: 84.8% for the HBV-positive group vs. 82.3% for the HBV-negative group, *p* = 0.47) or graft loss (3-year graft survival: 77.9% for the HBV-positive group vs. 79.7% for the HBV-negative group, *p* = 0.72). Similar findings were reported using data from the China Liver Transplant Registry [66]. Among 259 recipients of HBsAg-positive allografts and 259 matched recipients of HBsAg-negative allografts, the investigators observed comparable 3-year survival rates (60.4% vs. 69.1%, respectively, *p* = 0.062). These studies highlight the potential for HBV-positive allografts to safely increase the available donor pool without compromising outcomes. Prior to transplant, a thorough histologic evaluation of the donor’s liver was performed to confirm the absence of significant fibrosis. Additionally, grafts from donors with known HDV co-infection should be discarded given the lack of effective therapies for chronic HDV infection [70].

Transplantation with these HBV-positive liver allografts almost invariably results in chronic HBV infection in the recipient, making long-term suppression with NA therapy essential. High barrier-to-resistance NAs, such as ETV, TDF, and TAF, should be prioritized. If the recipient does not respond adequately to first-line NA therapy, additional strategies to achieve complete viral suppression should be explored, including HBV DNA polymerase sequencing, to identify resistance mutations and tailor therapy accordingly. The role of HBIG is likely limited in recipients of HBsAg-positive liver allografts, as HBIG only prevents HBV infection of the allograft but does not provide any therapeutic benefits once HBV infection has already occurred, as is the case with HBV-positive donor allograft transplant.

Another important consideration in the recipient of an HBsAg-positive graft with resultant chronic hepatitis B infection is the risk of de novo HCC in these immunosuppressed individuals. Given that HBV is a pro-oncogenic pathogen, there is concern for an increased risk of HCC in recipients of HBV-positive allografts. While the absolute risk of HCC in this transplant population is not well defined, adequate viral suppression with NAs has consistently been shown to significantly reduce the risk of HCC in the immunocompetent population [71,72]. Given the increased risk of HCC due to the use of immunosuppressants in a post-OLT population, it is important to consider HCC screening for all recipients of HBV-positive allografts with liver imaging (e.g., ultrasound, computed tomography, or magnetic resonance imaging) and alpha-fetoprotein (AFP) every six months. This is particularly critical for recipients of grafts from older donors of Asian or African descent, who may carry an elevated baseline risk for HCC.

Nonhepatic transplant recipients of HBsAg-positive allografts

The use of nonhepatic organs from HBsAg-positive donors has historically been considered marginal due to the risk of HBV transmission. However, nonhepatic allografts can also be utilized to improve the donor pool and transplant access [73,74]. While the liver is the main source of HBV transmission, HBV remains present in the circulation and lymphocytes, presenting a lower but still notable risk of de novo HBV infection [75].

Preliminary data have demonstrated satisfactory outcomes when recipients are treated with antiviral therapy, with or without HBIG prophylaxis. Delman et al. found that among fifty-six kidney transplant recipients of HBV NAT-positive donors, nine (16.7%) developed de novo HBV infection and active viremia [67]. All nine patients achieved HBsAg clearance after NA therapy with ETV and none developed any HBV-related complications. Graft and recipient survival rates were excellent, suggesting that effective antiviral therapy can mitigate the risks associated with HBV-positive allografts. Tuncer et al. observed no cases of de novo HBV infection among 35 HBV-immune recipients of kidneys from HBsAg-positive and HBV DNA-negative living donors [76]. Of interest, neither HBIG nor NA was used prophylactically for the patients in this study. These findings suggest that natural HBV immunity may mitigate the risk of DNH in such cases. Finally, Yin et al. reported on 105 recipients of kidneys from HBsAg-positive, HBeAg-positive, and HBV DNA-positive donors [74]. Outcomes were compared with those of recipients of kidneys from HBsAg-negative/anti-HBc-positive donors, and graft and survival rates were found to be similar. Four (3.8%) recipients developed an HBsAg-positive de novo infection; all four patients had subsequent conversion to HBsAg-negative status following NA therapy. There is limited data on thoracic organ transplantation involving HBsAg-positive donors, but satisfactory outcomes have also been reported. In a meta-analysis by Yost et al. of heart transplant recipients, 1 of 11 recipients of HBsAg-positive donors developed de novo HBV infection post-transplant, which was managed with lamivudine.

The optimal management of recipients of HBsAg-positive donors is unknown, but the risk of de novo HBV infection likely exceeds that of nonhepatic, SOT recipients of anti-HBc-positive allografts, and the prophylactic strategy should be tailored accordingly [54,55]. Prophylactic NA therapy (combined with HBIG therapy for non-immune recipients) should be considered for all nonhepatic SOT recipients. Routine laboratory monitoring should include assessments of HBsAg and HBV DNA, and liver function tests should be considered at least every three months to detect any signs of HBV transmission.

## 8. Conclusions

In conclusion, effective management of HBV reactivation and donor-related HBV transmission in immunosuppressed and solid organ transplant populations hinges upon a risk-stratified approach tailored to HBV serostatus, the type of immunosuppression, and patient-specific factors, including HBV status of donors and patient ability to adhere to long-term monitoring, especially if not prophylactically treated. The use of high-barrier NA therapies such as ETV, TDF, or TAF has proven instrumental in mitigating the risks of HBV reactivation and attendant complications in immunosuppressed patients from both non-transplant and transplant settings. For transplant recipients, the use of HBV-positive allografts, while historically limited, has emerged as a viable strategy to expand the donor pool, achieving recipient and graft survival rates comparable to those of HBV-negative allografts when appropriate prophylaxis is employed. The use of these HBsAg-positive allografts merits further investigation, including an assessment of the optimal prophylactic strategies and the long-term risk of HCC development.

## Figures and Tables

**Table 1:** HBV reactivation risk according to HBV serologic status and type of immunosuppression *.

HBV Status	HBsAg Positive (CHB)	HBsAg Negative/anti-HBc Positive (Latent HBV)
High risk (>10%)	- Anti-CD20 monoclonal antibodies: rituximab, ofatumumab, obinutuzumab - Moderate to high dose corticosteroids (prednisone ≥ 10 mg/day for ≥4 weeks) - Anthracycline derivatives - TNF-alpha inhibitors - Immune checkpoint inhibitors - HCV co-infection undergoing direct-acting antiviral therapy - Tyrosine-kinase inhibitors including imatinib and nilotinib	- Anti-CD20 monoclonal antibodies: rituximab, ofatumumab, obinutuzumab
Moderate risk (1–10%)	- Calcineurin inhibitors - Low-dose corticosteroid use (prednisone < 10 mg/day ≥ 4 weeks) - Cytotoxic chemotherapy	- Moderate to high dose corticosteroids (prednisone ≥ 10 mg/day for ≥4 weeks) - Anthracycline derivatives - Cytotoxic chemotherapy - Calcineurin inhibitors - Tyrosine-kinase inhibitors including imatinib and nilotinib
Low risk of reactivation (<1%)	- Antimetabolites such as azathioprine, 6-MP, methotrexate - Short-term corticosteroids (<1 week) of any dose - Intra-articular steroid injections	- TNF-alpha inhibitors - Antimetabolites, azathioprine, 6-MP, MTX - Low-dose corticosteroids (prednisone <10 mg) - Short-term corticosteroids (<1 week) of any dose - Intra-articular steroid injections - HCV co-infection undergoing direct-acting antiviral therapy

* Adapted with permission from systematic review and guidelines [3,30].

**Table 2:** HBV recurrence or de novo infection risk according to recipient and donor HBV profile for liver transplant recipients.

Donor HBV Status	Recipient HBV Status	Suggested Management	Rationale for Management
HBV negative			
	HBsAg positive	- High-barrier NA - HBIG if high risk for reactivation, including detectable HBV DNA at transplant, NA resistance, non-adherence, co-infection with HIV and/or HDV	Highly effective in preventing HBV recurrence in transplanted allograft from extrahepatic viral reservoirs
	Anti-HBs negative/anti-HBc positive	No prophylaxis needed; on-demand NA	Low risk for recurrence as the native allograft is replaced
HBsAg negative, anti-HBc positive			
	Prior infection with immunity HBsAg negative, anti-HBs positive, anti-HBc positive	No prophylaxis needed; on-demand NA	Low risk for de novo HBV infection, estimated risk ~1% without prophylaxis
	HBV naïve, immunity via vaccination HBsAg negative, anti-HBs positive, anti-HBc negative	High-barrier NA	Elevated risk for de novo HBV infection, estimated risk ~13% without prophylaxis
	Prior infection without immunity HBsAg negative, anti-HBs negative, anti-HBc positive	High-barrier NA	Elevated risk for de novo HBV infection, estimated risk ~10% without prophylaxis
	HBV naïve and without immunity HBsAg negative, anti-HBs positive, anti-HBc negative	High-barrier NA	Very high risk for de novo HBV HBV infection, estimated risk ~47% without prophylaxis
HBsAg positive or HBV DNA positive			
	Any HBV status	High-barrier NA	NA therapy is indicated for reactivation and HBV-related complications as a transplant allograft with CHB infection

HBIG: HBV immune globulin; HIV: human immunodeficiency virus; HDV: hepatitis D virus; NA: nucleos(t)ide analog; CHB: chronic hepatitis B.

**Table 3:** HBV recurrence or de novo infection risk according to the recipient and donor HBV profile for nonhepatic solid organ transplant recipients.

Donor HBV Status	Recipient HBV Status	Suggested Management	Rationale
HBV negative			
	HBsAg positive	High-barrier NA	Prevent HBV reactivation in the setting of immunosuppression
	Anti-HBs negative/anti-HBc positive	High-barrier NA	Prevent HBV reactivation in the setting of immunosuppression
HBsAg negative, anti-HBc positive			
	HBV immune Anti-HBs positive Anti-HBc any status	No prophylaxis needed; on-demand NA	Low risk of de novo HBV infection (<1%)
	HBV non-immune Anti-HBs negative Anti-HBc any status	High-barrier NA	Prevent de novo HBV infection
HBsAg positive or HBV DNA positive			
	HBV immune Anti-HBs positive Anti-HBc any status	High-barrier NA	Prevent de novo HBV infection
	HBV non-immune Anti-HBs negative Anti-HBc any status	High-barrier NA + HBIG	Prevent de novo HBV infection

HBIG: HBV immune globulin; NA: nucleos(t)ide analog.
